# Thermal imaging reveals social monitoring during social feeding in wild chimpanzees

**DOI:** 10.1098/rstb.2021.0302

**Published:** 2022-09-26

**Authors:** Claire Barrault, Adrian Soldati, Catherine Hobaiter, Stephen Mugisha, Delphine De Moor, Klaus Zuberbühler, Guillaume Dezecache

**Affiliations:** ^1^ Institute of Biology, University of Neuchâtel, Neuchâtel, Switzerland; ^2^ Budongo Conservation Field Station, Masindi, Uganda; ^3^ School of Psychology and Neuroscience, University of St Andrews, St Andrews, UK; ^4^ Centre for Research in Animal Behaviour, University of Exeter, Exeter, UK; ^5^ Université Clermont Auvergne, LAPSCO CNRS, Clermont-Ferrand, France

**Keywords:** *Pan troglodytes*, social cognition, audience effects, skin temperature, social ecology

## Abstract

Understanding the affective lives of animals has been a long-standing challenge in science. Recent technological progress in infrared thermal imaging has enabled researchers to monitor animals' physiological states in real-time when exposed to ecologically relevant situations, such as feeding in the company of others. During social feeding, an individual's physiological states are likely to vary with the nature of the resource and perceptions of competition. Previous findings in chimpanzees have indicated that events perceived as competitive cause decreases in nasal temperatures, whereas the opposite was observed for cooperative interactions. Here, we tested how food resources and audience structure impacted on how social feeding events were perceived by wild chimpanzees. Overall, we found that nasal temperatures were lower when meat was consumed as compared to figs, consistent with the idea that social feeding on more contested resources is perceived as more dangerous and stressful. Nasal temperatures were significant affected by interactions between food type and audience composition, in particular the number of males, their dominance status, and their social bond status relative to the subject, while no effects for the presence of females were observed. Our findings suggest that male chimpanzees closely monitor and assess their social environment during competitive situations, and that infrared imaging provides an important complement to access psychological processes beyond observable social behaviours.

This article is part of the theme issue ‘Cognition, communication and social bonds in primates’.

## 1. Introduction

How are the affective lives of chimpanzees and other primates shaped by socio-ecological context? In recent decades, much has been learned about what information primates process when navigating their social environment [1], including information on others' physical condition [2,3], rank [4,5], their own as well as others’ social relationships [6–8], and others' knowledge states [9–12]. This understanding has been developed from studies using both systematic observations (e.g. [7]) and experiments (e.g. [3]), typically based on observable behavioural markers, such as gaze (e.g. [10,13]), vocalization (e.g. [9,13–15]) or movement (e.g. [8]).

Often however, primates and other animals do not show observable responses to key social events, despite evidence that events are perceived, processed and often have future behavioural implications [1,16]. Primates continuously and closely monitor their social environment [17]. Affective responses play a crucial role in fostering social relationships (e.g. for emotional book-keeping: [18]; for the neuropeptide theory of social bonding: [19]), and in navigating social challenges, such as aggression or the threat thereof [8]. However, these important affective responses can be challenging to investigate, as they do not always include direct, easily observable, behavioural correlates.

Nevertheless, the role affective processes play in primate social lives has been gradually uncovered using methods such as hormonal profiling (e.g. oxytocin metabolites [20–23], corticosteroids [22,24–27] or pupillary dilatation [28]).

More recently, infrared thermography, a highly versatile technique increasingly used with both captive [16,29–36] and wild primates [37–40], has become part of the methodological toolkit [41]. Thermal imaging is particularly useful as it can offer a near real-time snapshot of the affective life of animals, providing an indirect measure of arousal [33,41]. More precisely, thermal imaging is a contact-free method that can assess the surface temperature of bodies through their wavelength and electromagnetic radiation [41–43]. Recent technological progress has made this technique sensitive to changes in peripheral blood flow [43,44], although other physiological processes, such as metabolic heat production, can also affect skin surface temperature [41]. Changes in blood flow are part of adaptive response mechanisms, measurable in temperature changes in the peripheral body parts, such as the perinasal area [30] or the tail [45]. Previous studies have demonstrated that situations likely to be perceived as stressful by an animal can lead to significant drops in nasal temperatures [30,36,39,44]. The general assumption is that changes in peripheral blood flow reflect changes in activity of the autonomic nervous system and that these changes can be used as a way to assess an animal's ‘internal perspective’ (*sensu* [1,16]). For example, they have been used to show that chimpanzees respond to relevant social events, such as physical aggression seen in others [30,39], and/or participation in prosocial events, such as mutual grooming [39].

In the current study, we used thermal imaging to evaluate arousal levels in chimpanzees engaged in feeding while in the company of others, with the intention of uncovering whether individuals show otherwise non-observable responses to the presence of others, depending on the type of resource at stake, and the nature of their social relationship to those around them. Social monitoring is a putative function for well-documented audience effects [46,47] in which the presence of others impacts both cognitive and affective functions [48]. Audience effects have received substantial attention, both in the human and non-human animal literature (humans [48,49], primates [50–52], other species [53–55]). In humans, the presence of an audience considerably impacts our cognitive processes, such that the mere presence of others affects our attentional resources [48,56]. Audience effects also occur in non-human animals, and one hypothesis is that audiences which increase the risk of aggression or interference with one's own activities lead to increased social monitoring and arousal [46,47,49].

In chimpanzees, social feeding carries an elevated risk of aggression owing to resource competition [57–61]. However, the perceived threat can vary dramatically from one feeding event to the next, in relation to changes in food patch size, monopolizability and desirability of food items, as well as nearby group members [56,62–64]. A particularly valuable food resource for chimpanzees is meat, usually the result of lengthy and socially coordinated hunting behaviour that can involve a considerable number of individuals jointly attacking a group of monkeys or other prey [65–68]. If successful, the carcass can be monopolized by one individual and defended against competitors, who may use various degrees of coercion to induce sharing [63], although meat may also be shared freely with social partners [7]. As a result, the acquisition and consumption of meat is probably socially more complex and associated with higher levels of competition than other, more commonly consumed and less easily monopolized foods, such as small- and medium-sized figs that can be harvested from large trees [68] and are typically of more moderate energetic value [69].

Chimpanzees, like many social animals, live in groups with individualized relationships, characterized by dominance, kinship, and social bonding [70,71]. As feeding typically takes place in the presence of others, the size and composition of the nearby audience is likely to impact how individuals perceive the social competition associated with a feeding event. In particular, we expect it to be impacted by the social context. For example, trying to retrieve a fruit near a dominant non-bonded group member is probably perceived as more stressful than doing so from near a social partner [7,23,56,63], owing to differences in the risk of aggression. Importantly, these changes in stress response may not be easily directly observed, whether by human observers or conspecifics, from whom it may be advantageous to conceal them [72].

We used infrared thermography to assess how wild chimpanzees' arousal changes during different types of feeding events (meat versus figs), in the presence of male and female group members, and with or without the presence of dominant individuals or social partners. Based on previous research with chimpanzees [30,31,37,39], we focused on temperature differences on the surface of the nose, a body part that reacts with measurable shifts in skin temperature in response to various types of external events [44], including perceived aggression [30,39]. We did not monitor nasal temperature across time (as locomotion is likely to affect skin temperature), but as single independent events. For this reason, we analysed relative differences across variables of interest, namely food type and audience composition. We predicted lower nasal temperatures for larger audiences, particularly when feeding on meat versus figs. More specifically, we predicted lower temperatures for large male audiences, who are not only competitors but also potential aggressors [73]. Similarly, we predicted lower temperatures in the presence of more dominant individuals. We predicted higher temperatures in the presence of social partners, who are less likely to aggress and more likely to support the subject during aggression, providing a potential social buffer in the context of competition. We expected the effects to be larger when feeding on meat as compared to figs. We control for the impact of mating competition as it is a social factor known to affect chimpanzee arousal levels [74,75] (in particular around nutritious food sources [76]).

## 2. Methods

### (a) Field-site

Data collection were conducted in the Waibira community of the Budongo Central Forest Reserve, in north-western Uganda, a moist, semi-deciduous tropical rain forest covering 428 km^2^ at an altitude of 1100 m, between 1°35′ and 1°55′ N and 31°08′ and 31°42′ E [77]. Habituation of the Waibira community started in 2011 and most members of the community were well-habituated, including all adult males and all central adult females [78]. At the time of the study, the community was composed of approximately 100 named individuals. Some peripheral adult females and their dependent offspring, as well as some young infants remained unnamed (all are individually recognized and, when seen, interact socially with named individuals as part of the same community), giving an estimated total community size of around 120 individuals.

### (b) Data collection

Data were collected between August 2018 and January 2019 by C.B. and S.M., using focal animal sampling [79] approximately during 07.00 to 17.00 focal follows for approximately 5.5 days a week. Focal animals were 19 adult individuals, nine females (15–37 years old) and 10 males (15–42 years old) (electronic supplementary material, table S1). The focal individuals were chosen because of their tolerance to the presence of observers within 10 m. Every day, one focal individual was chosen amongst the individuals encountered in the forest. No focal individual was followed for two consecutive days, and sampling time was balanced across individuals. Each focal individual was observed on average for approximately 13 h (range 9–15 h). Behavioural data were entered on a portable device (Runbo F1) using a custom built-in interface created with Cybertracker (v. 3.389).

Thermal images were largely taken from focal individuals but, when this was impossible, they were opportunistically taken from other party members. ‘Subjects’ hereafter refers to individuals being photographed. Thermal images were taken using a TESTO thermal imager model 881–2. The camera sensor possesses a spectral range from 8 to 14 µm and a thermal sensitivity of less than 50 mK at 30°C. Emissivity was set at 0.98, a value used for chimpanzee thermal imaging [37,38]. A telephoto lens (9° × 7°/0.5 m) was attached to the camera at all times. Images were taken during feeding events from a distance of approximately 7 m (range 2–15 m) whenever possible to minimize the effect of distance on temperature reading [80], when subjects were feeding either on the ground or in trees (up to a maximum height of 2 m), and only when they were engaged in social feeding behaviour (i.e. feeding in the presence of at least one other subadult or adult individual within 10 m). Subjects were recorded when feeding on meat (*Colobus guereza*, *Cercopithecus mitis*, *Cephalophus natalensis*), or on figs (here, largely on *Ficus sur*—a tree that produces large volumes of small figs distributed across its branches and trunk and of intermediate value, with respect to its level of sugar compared to other fig species [69]; and, infrequently, on *Ficus exasperata*).

Subjects had to be stationary for at least 1 min prior to taking images (to avoid the effects of locomotion on nasal temperature [30]), not exposed to direct sunlight, and when there was a direct and unobstructed line of sight. Once all of these conditions were met, we took as many pictures as possible (on average, one every 5 s). For each thermal image, we recorded the following information about adult and subadult individuals present within 10 m: the number of males and females, the number of social partners (defined below) and the dominance distance between the subject and the highest-ranking individual (defined below). Thermal data collection was interrupted as soon as the individual was not engaged in feeding or when the subject moved more than 5 m from the initial location. To control for environmental confounds, humidity (which affects emissivity) and ambient temperature (which affects surface temperature) were recorded every 15 min using a portable digital thermometer and hygrometer (HTC-1 LCD).

### (c) Data analysis: measurement of infrared thermography data

**Figure 1.**
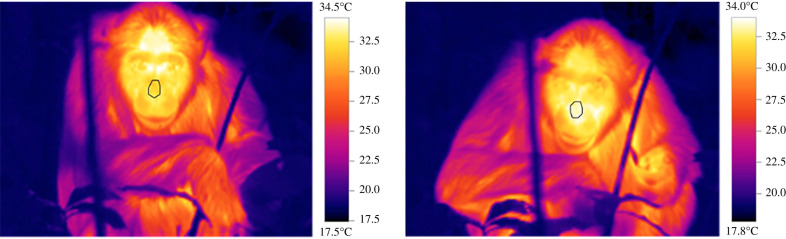
Examples of thermal images from a chimpanzee feeding on *Ficus sur*. The drawing around the nasal area represents the region of interest. (Online version in colour).

Given that chimpanzee skin temperature can change rapidly (within about 10–20 s [37]) we considered two thermal images as statistically independent if they were separated by greater than 1 min. As the image angle can affect readings [81], we only included images if both eyes of the subject were clearly visible (maximum angle of 45°; figure 1). We extracted temperature measurements from the nasal area, using the TESTO IRSoft analysis software (v. 4.0.5; figure 1). Because air flow entering and exiting the nose may create artefacts in temperature measurements, we manually drew a six-sided diamond-shaped polygon around the nasal area, avoiding the nostril area, and extracted the mean temperature. Intra-rater reliability was performed on *n* = 89 images (71% of the dataset used for analysis), which showed a high degree of agreement (intraclass correlation coefficient = 0.99).

### (d) Data analysis: social correlates of thermal images

#### (i) Social partners and dyadic composite sociality index

The dyadic composite sociality index (DSI) is calculated as the sum of the duration of time a dyad spent grooming and a dyad spent in proximity, divided by the mean recorded duration of that behaviour, and then divided by the number of behaviours. We computed DSI for all dyads including a focal, based on positive social interactions that occurred between the two individuals. Both adults and subadults were considered as well as same-sex and mixed dyads. The DSI scores were calculated as described in [82], but using only grooming duration (with a distinction made between reciprocal and unidirectional grooming, the former being attributed to both individuals, with duration counted as double) and time spent in proximity (all individuals within 5 m of the focal were considered to be ‘in proximity’). DSI values ranged from 0 to infinity, with dyads with a DSI greater than 1 having a better than average social relationship relative to all recorded social relationships. For each of the subjects, we determined three social partners, corresponding to the three individuals with which the subject exhibited the highest DSI values (among the DSI values > 1) [79], excluding dependent offspring (see the electronic supplementary material, table S2). Number of social partners within 10 m was therefore defined as the number of individuals within 10 m to the subject that were among the top three social partners of the subject.

#### (ii) Dominance distance

We computed Elo ratings (dominance ranks) for all individuals based on unidirectional pant-grunts (a submission signal in chimpanzees [83]) collected ad libitum during the study period, using the ‘EloRating’ package (v. 0.46.11 [84,85]). The individual Elo ratings at the end of the study period were used as the dominance rank of the individual throughout the study period and applied to all recorded events. No obvious rank changes were observed during the study period, suggesting that the Elo-scores assigned to each individual were reliable estimates of their relative positions within the dominance hierarchy. We calculated dominance distance as the difference between the Elo rating of the highest-ranking individual within 10 m of the subject and the Elo rating of the subject. Positive values of dominance distance therefore indicate that the subjects were submissive to at least one individual in their audience, whereas negative dominance distance values indicated that the subject outranked all individuals in their audience.

#### (iii) Control for mating competition

We included the number of females in oestrus within 10 m of the subject as a proxy for mating competition.

### (e) Statistical analysis: models

Statistical analyses were performed in R (v. 4.1.2 [86]) using R Studio (v. 2021.9.1.372 [87]).

We built general linear mixed models (GLMM) using the ‘lmer’ function of the ‘lme4’ package (v. 1.1.27.1 [88]). Analyses were performed on *n* = 124 observations from 19 individuals (10 adult males, nine adult females), based on social feeding on ‘meat’ (here: meat of *Colobus guereza*, *Cercopithecus mitis*, *Cephalophus natalensis*) (*n* = 55 observations) or ‘figs’ (here: *Ficus sur, Ficus exasperata*) (*n* = 69 observations).

The dependent variable was nasal temperature, which was transformed to reduce skewness ((datapoint - min(of all datapoints)^2^) and *z*-scored. The predictors were: food type (meat or figs); number of males (within 10 m of the subject); number of females (within 10 m of the subject); number of social partners (number of top three social partners of subject within 10 m of the subject) and dominance distance (Elo rating of highest-ranking individual within 10 m of the subject − Elo rating of subject). All predictors were *z*-scored. We also included three control variables: ambient temperature, expressed in *Celsius; ambient humidity, expressed in % of humidity in the air and number of females in oestrus within 10 m as a proxy for mating competition. All control variables were *z*-scored.

Colinearity checks were carried out using the function ‘vif’ of the package ‘car’ (v. 3.0.1, [89]). They indicate no colinearity issues (all variance inflation factors (VIFs) value below 1.9). Our sample size was within the range required for analysis using GLMM [90] (sample size ≥ 104 + no. predictors; sample size ≥115 in our case).

The full model comprised interactions between the predictor food type and all other predictors, as well as the two control variables and the identity of the subject as a random factor. We confirmed that the residuals were normally distributed and homogenous by inspecting a scatterplot and quantile-quantile plot of the residuals in function of the fitted values, and that no influential data points were present by excluding data points one at a time from the dataset and checking model estimates.

Before assessing the significance of the predictors, we tested our full model including all interactions against a null model (including only the control variables and random effect) using the function ‘Anova’ of the package ‘car’, to test whether the predictors improved model fit. We also reran the same full model, additionally including a random effect for thermal images taken within a 30 min time period, to ensure the results were robust to temporal auto-correlation. *p*-values were extracted using the function ‘Anova’ from the package ‘car’ (v. 3.0.1, [89]).

Figures were created using the packages ‘ggplot2’ (v. 3.3.5 [91]) and ‘ggpubr’ (v. 0.4.0 [92]).

## 3. Results

Our full model was a better fit than the null model (likelihood ratio test: *χ*^2^_9_ = 700.98, *p* < 0.001) and revealed a significant interaction between food type and the number of males within 10 m, the number of social partners within 10 m and the dominance distance to the highest-ranking individual within 10 m. We found no evidence for an effect of the interaction between food type and the number of females within 10 m, nor for an effect of the number of females independently after rerunning the model without the interaction term (table 1; electronic supplementary material, table S3). These results were robust to the inclusion of a random effect term for data points collected within the same 30 min time period (see the electronic supplementary material, table S4). Note also that, as expected, ambient temperature was a strong predictor of nasal temperature. We found no evidence for a significant effect of number of females in oestrus, used as a proxy for mating competition (table 1).

**Table 1.** Interaction effect of food type and audience composition on nasal temperature. (The reference level for ‘food type’ is ‘meat’. Significant results are in bold. Interactions are noted with an asterisk.)

term	estimate	s.e.	*t*-score	*p*-value
intercept	−4.435	2.407	−1.842	0.065
food type	0.227	0.222	1.019	0.308 **^b^**
**number of males**^a^	**0**.**373**	**0**.**109**	**3**.**421**	**0.001^b^**
number of females**^a^**	0.068	0.150	0.451	0.652**^b^**
number of social partners**^a^**	−0.312	0.182	−1.704	0.088**^b^**
dominance distance**^a^**	0.118	0.119	0.993	0.321**^b^**
**ambient temperature^a^**	**0**.**172**	**0**.**068**	**2**.**546**	**0**.**011**
ambient humidity**^a^**	−0.015	0.015	−1.003	0.316
**number of oestrus females^a^**	−**0**.**085**	**0**.**083**	−**1**.**023**	**0**.**306**
food type * number of males^a^	−**0**.**591**	**0**.**208**	−**2**.**840**	**0**.**005**
food type * number of females^a^	0.056	0.190	0.303	0.762
food type * number of social partners^a^	**0**.**591**	**0**.**228**	**2**.**596**	**0**.**010**
food type * dominance distance^a^	−**0**.**380**	**0**.**171**	−**2**.**221**	**0**.**026**

^a^*z*-scored to allow comparison of model estimates across predictors.

^b^Note that main effects have limited interpretative value when interactions are significant.

**Figure 2.**
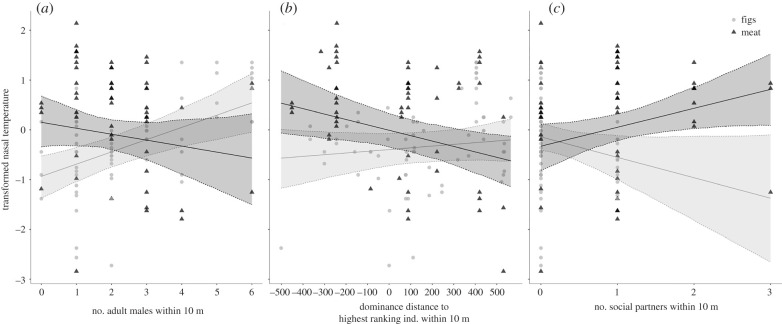
Interaction effect of food type and audience composition on nasal temperature (figs, grey circles; meat, black triangles). The fitted model lines (figs, light grey; meat, dark grey) are presented with 95% confidence bands for the fitted values (95%). (*a*) Interaction effect between the number of males within 10 m and food type on nasal temperature; (*b*) interaction effect between the dominance distance to the highest-ranking individual within 10 m (Elo rating of highest-ranking individual within 10 m of the subject − Elo rating of subject) and food type on nasal temperature; and (*c*) interaction effect between the number of social bond partners presents within 10 m and food type on nasal temperature.

Nasal temperature while feeding on meat was lower for higher numbers of males within 10 m (figure 2*a*). The opposite pattern was found for figs, with higher nasal temperature for higher numbers of males within 10 m (figure 2*a*).

The model also revealed an interaction between food type and dominance distance. When feeding on meat, nasal temperature was lower for higher dominance distances to the highest-ranking individual within 10 m (figure 2*b*). We found no clear effect of dominance distance when feeding on figs.

Finally, the model showed an interaction between food type and the number of social partners within 10 m. The nasal temperature while feeding on meat was lower for fewer numbers of social bond partners present within 10 m. An opposite effect was observed for figs (figure 2*c*), although the effect should be treated with caution because of a limited number of data points for a larger number of social partners within 10 m.

Table 1 recapitulates the model results. Note that the values for the main terms are presented but, because of the presence of significant interactions, the interpretation of the individual main effects is limited to their effect, keeping the interacting effect at its average [93].

## 4. Discussion

In the human literature, audience effects are well documented, such that the mere presence of others is known to affect one's attentional resources and level of arousal [48,49,53,56]. One putative function of such audience effects is the ‘social monitoring’ of others to best prepare oneself for possible intrusion in one's own activities [46,49], including potential aggression. Social feeding, because it carries an elevated risk of aggression, is an ideal context in which to evaluate this hypothesis in chimpanzees [57,94].

To date, animal behaviour researchers were largely limited to the documentation of easily observable behaviours, such as shifts in gaze, or in relative position. Recently, infrared thermal imaging has gained prominence as an important new tool, providing a simple and non-invasive method to assess otherwise hidden physiological responses that can imply real-time cognitive processing of social interactions that would be undetectable with traditional methods [41,44].

We used infrared thermography to study the role of audience effects during feeding events in wild chimpanzees, with the hypothesis that chimpanzees passively monitor the audience when valuable resources are at stake. Our results revealed differences in nasal temperature related to the food resource, the audience size, and the audience composition (dominance and social partners). Specifically, nasal temperature of individuals feeding on meat were lower for contexts marked by higher competition, i.e. both when more males were present within 10 m, and when dominance distances between a lower-ranking subject and the most dominant individual in the party were large. These findings are consistent with elevated levels of stress when surrounded by dominant individuals and feeding on meat, a more easily monopolized and important resource [7,63]. Our results also suggest that social partners may have acted as a buffer (*sensu* [95]), with higher nasal temperature for individuals with more social partners within 10 m (relative to others with fewer social partners around) when feeding on meat.

This pattern of results is consistent with the findings of reduced temperature in peripheral areas of animals' bodies (such as the nose, or the tail) owing to blood redirection away from areas vulnerable to significant blood loss [45] in social situations likely to be perceived as stressful [30,39,41,44]. These findings support the hypothesis that social feeding carries an elevated risk of physical aggression and stress that is likely to be mitigated by the presence of social partners [96] and the prospect of receiving coalitionary support in case of escalation [8,97,98]. Interestingly, we found the opposite patterns of results for figs, with higher levels of nasal temperature for larger numbers of males present within 10 m, and for lower numbers of social partners present within 10 m. While figs are an important food resource [69,71], the small-sized *Ficus sur* (which accounted for the majority of observations of feeding on figs in our sample) represent a less competed-for and less monopolizable food resource than meat. In a large community such as Waibira, with an unusually large number of independent males, having more male and high-ranking individuals in a party could perhaps decrease the chance of aggression. The presence of so many males, and therefore of potential allies for whomever is threatened, makes any use of aggression a risky strategy. If this is the case, then when feeding on small-medium sized figs, a much more widely available and less easily monopolized food than meat and thus one less worth competing over, having more males and high-ranking individuals present could represent a lower risk environment than a smaller party with just one or two large dominant individuals present.

The presence of females in oestrous, a characteristic of the environment which is probably conducive to escalated risk of aggression [75], did not impact skin temperature. A possible reason is that there is a trade-off between mating and feeding in terms of time budget, such that feeding may be already reduced when mating opportunities are available (as seen in [99]) and mating opportunities thus had a reduced impact on the limited time left for feeding.

The mere presence of others can affect cognitive, behavioural and emotional responses (see [49] for a review). Several hypotheses have been discussed as to why this is the case. One hypothesis (referred to here as the ‘social monitoring’ hypothesis) is based on the arousal levels generated by the presence and proximity of others [53,100], among which are potential competitors for resources and/or individuals whose behaviour remains uncertain and can represent a physical threat to the subject [46,47], but who can also represent protection from non-social partners and provide social support [21,101]. In chimpanzees, social feeding can be characterized by an elevated risk of aggression [58], which makes monitoring of audience members and their social relationships particularly important. Audience effects are particularly expected for high-value food, such as meat, relative to more spatially dispersed, more abundant and less easily monopolized feeding sources, such as figs. This said, even in the absence of overt physical competition over food (as manifested by, for example, physical aggression or milder harassment, such as persistent begging gestures towards the individual in possession [63,102]), the physiological effects of arousal observed might also reflect an effect of the presence of others [48,53].

While discussion remains over the exact source of body surface temperature shifts [41], some consensus exists over the valid use of infrared thermography to infer shifts in blood flow caused by the activation of the autonomic nervous system [44], with a decrease in nasal temperature when individuals' are exposed to stressful events [30,36,39,44]. Although it is tempting to interpret the observed differences in temperature as reflecting absolute levels of arousal, no time series were collected here, preventing interpretation of our data as indicating shifts in skin temperature relative to a known baseline or changes occurring after a specific stimulus (unlike in e.g. [30,37]). We therefore interpret skin temperature profiles strictly as ‘relative’ differences across various audience sizes and composition, with lower nasal temperature indicating relatively lower blood flow in peripheral regions of the body, a pattern typically associated with the experience of negative emotions [44].

While our findings are strongly suggestive, they remain—like much of the thermal imaging field—preliminary. We would benefit substantially from much larger datasets that allow analysis both within individuals, and between a larger number of individuals with a greater range of social relationships. In future work, longitudinal comparisons may provide a means to explore variation in coping-style across individuals, a fundamental aspect of how other species (including humans) vary in their individual responses to stressful events [103,104]. Future studies could also include complimentary physiological measurements (such as hormonal profiles, e.g. [30]) that could support and enrich our understanding of the effects being observed (e.g. help identify the physiological source of changes in arousal, for example the potential involvement of the ‘hypothalamus–pituitary–adrenal axis, as in [30]).

In summary, our study shows that wild chimpanzees are affected by the presence of audience in stressful situations, such as when feeding over potentially contested resources, and that audience effects in non-human apes include nuanced monitoring of their day-to-day social landscape.

## Data Availability

Data are available at osf.io/c5pku. Data are also provided in the electronic supplemenary material [106].
